# The Natural History of Hematemesis in the 21st Century

**DOI:** 10.7759/cureus.3029

**Published:** 2018-07-23

**Authors:** Leen Khoury, David A Hill, Melissa Panzo, Melissa Chiappetta, Sachin Tekade, Stephen M Cohn

**Affiliations:** 1 Research/surgery, Staten Island University Hospital/Northwell Health, Staten Island, USA; 2 Plastic and Reconstructive Surgery, Houston Methodist Hospital, Houston, USA; 3 Research, Staten Island University Hospital/Northwell Health, New York, USA; 4 Surgery, Staten Island University Hospital/Northwell Health, Staten Island, USA; 5 Surgery, Staten Island University Hospital, Queens Village, USA

**Keywords:** gastrointestinal bleeding, upper gastrointestinal hemorrhage, hematemesis, therapeutic endoscopy

## Abstract

Objective: Upper gastrointestinal (GI) bleeding occurs at a rate of 40-150 episodes per 100,000 persons per year and is associated with a mortality rate of 6%-10%. We sought to determine the need for therapeutic endoscopy or surgical interventions in patients with hematemesis and the association with blood transfusion requirements.

Methods: We queried the database of our large teaching facility for adult patients presenting with obvious upper GI hemorrhage (hematemesis) between 2014 and 2017. We evaluated the amount of blood transfusions administered and the need for operative, endoscopic or angiographic interventions.

Results: Eighty-one patients were admitted with hematemesis: mean age was 63 years old (range 21-103), 60% were male, and mean hemoglobin was 11.3 g/dL (range 3.6-15.6). Forty-one percent received blood transfusions with a mean of one unit transfused per patient (range 0-10); 9% received ≥ 3 units of packed red blood cells. Bleeding stopped spontaneously in 88% of patients and nine died. Forty-seven percent underwent inpatient endoscopy but only 6% underwent a therapeutic endoscopic intervention. No patient had a surgical or interventional radiologic procedure related to their GI bleed.

Conclusion: Upper GI bleeding rarely requires operative or interventional radiologic intervention. Blood transfusions were not predictive of the need for therapeutic endoscopic intervention which was required in only 6% of patients.

## Introduction

Gastrointestinal (GI) hemorrhage results in approximately 300,000 hospital admissions in the United States annually. Upper GI bleeds occur at a rate of 40-150 episodes per 100,000 persons per year, with a mortality rate of 6%-10% [1].

With the advent of new anti-ulcer medications, the diseases associated with upper GI bleeding are now better controlled and the resulting complications associated with these illnesses appears to be in a state of decline. In the United States, the incidence rates for hospital discharges with the diagnosis of peptic ulcer disease decreased from 1,400 per 100,000 people in 1979 to approximately 700 per 100,000 in 2004 [2]. Upper GI bleeding decreased from 78.4 to 60.6 per 100,000 people between 2001 and 2009 and there has been a decrease in peptic ulcer bleeding from 48.7 to 32.1 per 100,000 [3].

With all of these changes regarding diseases associated with upper GI bleeding, we sought to determine the need for therapeutic endoscopy or surgery in patients with hematemesis and the association with blood transfusion. We hypothesized that the need for therapeutic interventions in patients presenting with hematemesis has decreased dramatically.

## Materials and methods

A retrospective chart review was performed which examined adult patients admitted to Staten Island University Hospital, a 714 bed teaching facility, with a diagnosis of upper gastrointestinal (GI) bleeding between 2014 and 2017. During that time, 318 patients were admitted to the hospital with a diagnosis of upper GI bleeding. Of these patients, eighty-one were included in our study as they presented with hematemesis (vomiting of bloody gastric fluid or had blood on nasogastric aspiration).

The endpoints of the study were: endoscopic, operative, or angiographic interventions performed and total blood transfusions administered. Therapeutic management (surgical, interventional radiology, endoscopic), transfusion requirements, mortality and length of hospital stay as well as basic demographic information were analyzed. We also studied the relationship between the amount of blood transfused and the types of interventions performed.

The Institutional Review Board of Northwell Health/Feinstein Institute provided protocol approval and a waiver of informed consent was obtained. Statistical analysis was performed utilizing student t-test to evaluate continuous variables. Non-continuous variables were evaluated using a Chi square analysis. Statistical significance was set at a value of p < 0.05.

## Results

Eighty-one patients were admitted with obvious upper GI bleeding (hematemesis). The mean age was 63 years old (range 21-103) with 60% being male. Average admission hemoglobin was 11.3 (range 3.6-15.6 g/dl) (Table 1).

**Table 1:** Patient demographics BP: blood pressure; HR: heart rate; Hgb: hemoglobin; PRBC: packed red blood cells; IR: interventional radiology; GI: gastrointestinal; N/A:not applicable.

		Total	Endoscopic Intervention	No Endoscopic Intervention	P-Values
Total		81	5	76	
Male Sex		61%	40%	62%	0.333
Anticoagulation Medication Use		38%	60%	37%	0.302
Antiplatelet Medication Use		10%	0%	11%	0.445
Combination of Antiplatelet and Antithrombotic Medication		5%	0%	5%	0.599
Anticoagulant or Antiplatelet Medication Reversal Agent Administered		9%	0%	9%	0.478
>3 units PRBC Transfused		7%	0%	8%	0.514
Systolic BP (mmHg)	Mean	125 (72-221)	145 (107-221)	123 (72-193)	0.073
HR (beats/minute)	Mean	87.7 (52-155)	107.2 (90-155)	86.4 (52-128)	0.008
Admission Hgb (g/dl)	Mean	11.3 (6-17.5)	9.1 (7.1-11.4)	11.4 (6-17.5)	0.079
Lowest Hgb (g/dl)	Mean	9.4 (3.6-15.6)	8 (6.3-9.6)	9.5 (3.6-15.6)	0.182
Transfusion Required		41%	60%	40%	0.366
Amount transfused (units of PRBC)	Median	0 (0-10)	1 (0-2)	0 (0-10)	0.922

In 38% of cases, patients were on antiplatelet or anticoagulation medications, (5% on both). Reversal agents were used in 18% of patients taking either of these types of medications.

Bleeding stopped spontaneously in 88% of patients without any intervention. Eleven percent of patients (n=9) died. Deaths were related to refusal of transfusions, requests for comfort care measures, withdrawal of care or other causes unrelated to GI bleeding. Three of these deaths were due directly to GI bleeding. One expiration was a patient with multiple medical comorbidities (hypertension, venous thrombosis and atrial fibrillation) on warfarin with an International Normalized Ratio (INR) of eight. This patient also had an active do not resuscitate and do not intubate order. Despite intravenous fluids, fresh frozen plasma, blood transfusion and vasopressors the patient rapidly deteriorated and expired. The second patient died as a result of massive hemorrhage from a gastric ulcer. The patient was initially admitted with a diagnosis GI bleeding, but left the hospital against medical advice. Three days later the patient returned to the hospital with continued upper GI bleeding, deteriorated, developed cardiogenic shock and was unable to be resuscitated. The third expiration was a patient whom the family requested comfort care measures only. After a month-long stay, the patient died on the day of discharge to hospice care.

**Figure 1:**
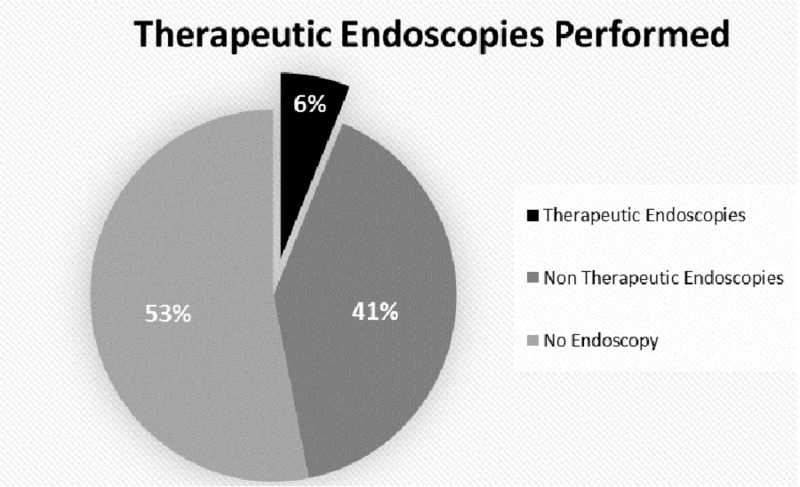
Endoscopies performed

Forty-seven percent of patients underwent inpatient endoscopy, but only 6% received a therapeutic endoscopic intervention (Figure 1).

Sixty percent of patients undergoing therapeutic endoscopy were on anti-platelet or anticoagulation medications. The average admission hemoglobin was 9.12 g/dl for those receiving a therapeutic endoscopy versus 11.43 g/dl in patients not undergoing an endoscopic intervention (p=0.078). Mean blood transfusion required for patients undergoing endoscopic intervention was one unit, but was 0 units in those not undergoing an intervention. No patients receiving ≥ 3 units of blood underwent a therapeutic intervention. No statistically significant differences in mortality, length of hospital stay or transfusion requirements were noted when those patients receiving a therapeutic endoscopy where compared with those who did not. No patients received a surgical or interventional radiologic procedure related to their GI bleed (Table 2).

**Table 2:** Endoscopic interventions versus no endoscopic interventions IR: interventional radiology; GI: gastrointestinal; N/A:not applicable.

		Total	Endoscopic Intervention	No Endoscopic Intervention	P-Values
Total		81	5	76	
Surgery Performed for GI Bleeding		0%	0%	0%	N/A
IR Procedure Performed		0%	0%	0%	N/A
Died		11% (9)	0% (0)	12% (9)	0.414
Length of stay (days)	Median	3 (0-27)	2 (1-8)	3 (0-27)	0.702

Only one patient underwent surgery, a craniotomy for evacuation of subdural hematoma. This patient presented to the hospital unresponsive and made no neurologic progress after their operation. The family withdrew care and the patient expired the next day.

In regards to blood transfusions: 41% had blood transfusions with a mean of one unit transfused per patient (range 0 to 10). Nine percent of patients received three or more units of packed red blood cells (PRBCs) and 7% received three or more units in the first 24 hours after admission. None of these patients requiring >3 units of blood had a therapeutic endoscopic, surgical or interventional radiologic procedure (Table 3).

**Table 3:** Comparison of >3 units of PRBC transfused versus BP: blood pressure; HR: heart rate; Hgb: hemoglobin; PRBC: packed red blood cells; IR: interventional radiology; GI: gastrointestinal.

		Total	>3 units PRBC (first 24 hrs)	<3 units PRBC (first 24hrs)	P-Value
Total		81	6	75	
Male Sex		61%	67%	60%	0.748
Systolic BP (mmHg)	Mean	125 (72-221)	105 (98-112)	126 (72-221)	0.054
Admission Hgb (g/dl)	Mean	11.3 (6-17.5)	8.6 (6.3-10.5)	11.5 (6-17.5)	0.014
Lowest Hgb (g/dl)	Mean	9.4 (3.6-15.6)	6.9 (6-8)	9.6 (3.6-15.6)	0.008
Transfusion Required		40.7%	100%	36%	0.002
On Antiplatelet or Anticoaguant Medication		44%	50%	44%	0.776
Endoscopy Performed		6%	67%	45%	0.314
Therapeutic Endoscopy Performed		6%	0%	7%	0.514
Surgery Performed for GI Bleeding		0%	0%	0%	-
IR Procedure Performed		0%	0%	0%	-
Any Therapeutic Intervention Performed		49%	0%	9%	0.434
Died		2.5%	0%	12%	0.368
Length of stay (days)	Median	3 (0-27)	7.5 (3-14)	3 (0-27)	0.127

## Discussion

GI bleeding continues to be an important medical issue causing approximately 67 per 100,000 hospitalizations annually in the US [4]. The average hospitalization costs are estimated to be between $3,402 to $5,632 for nonvariceal upper GI bleeding, and $6,612 to $23,207 for variceal related bleeding [5]. Fortunately, the hospitalization rates for the diagnosis of upper GI hemorrhage decreased by 21% from 2002 to 2012. The diagnosis of gastritis and peptic ulcer disease had the largest decrease in GI bleed related incidence, 55% and 30% respectively [4]. Perhaps this is in part due to the implementation of new medical therapies such as proton pump inhibitors or treatment of *Helicobacter pylori* [6]. Mortality rates have also improved by 28% [4]. This is likely due to improvements in resuscitation and advances in therapeutic endoscopic techniques [6]. The need for therapeutic interventions, however, appears to be diminishing. In our study, 47% of the patient population received an endoscopy but only 6% required a therapeutic endoscopic intervention. In 88% of patients, the bleeding stopped spontaneously.

In addition, we found no association between patients receiving more than three units of PRBCs and the need for intervention. These patients who required significant transfusions typically presented with lower hemoglobin values and received more transfusions. However, no patients who received more than three units of PRBCs required any therapeutic procedures, including surgical, interventional radiological or endoscopic.

This study is limited by a small population, despite a three-year review at a large urban hospital. We had limited information about endoscopies occurring immediately prior to admission or after discharge. Also, we had limited data on the timing from bleeding to endoscopy, but nearly 90% of bleeding stopped spontaneously.

## Conclusions

The need for surgical, interventional radiologic or therapeutic endoscopy is rarely required in the treatment of patients presenting with obvious upper GI bleeding. In 88% of patients with hematemesis, bleeding resolves spontaneously. There is no association between transfusion requirements and the need for a therapeutic intervention.

## References

[1] Kim BS, Li BT, Engel A (2014). Diagnosis of gastrointestinal bleeding: a practical guide for clinicians. World J Gastrointest Pathophysiol.

[2] Tielleman T, Bujanda D, Cryer B (2015). Epidemiology and risk factors for upper gastrointestinal bleeding. Gastrointest Endosc Clin N Am.

[3] Laine L, Yang H, Chang SC, Datto C (2012). Trends for incidence of hospitalization and death due to GI complications in the United States from 2001 to 2009. Am J Gastroenterol.

[4] Wuerth BA, Rockey DC (2018). Changing epidemiology of upper gastrointestinal hemorrhage in the last decade: a nationwide analysis. Dig Dis Sci.

[5] Adam V, Barkun AN (2008). Estimates of costs of hospital stay for variceal and nonvariceal upper gastrointestinal bleeding in the United States. Value Health.

[6] Pérez-Aisa MA, Del Pino D, Siles M, Lanas A (2005). Clinical trends in ulcer diagnosis in a population with high prevalence of Helicobacter pylori infection. Aliment Pharmacol Ther.

